# Ischaemic cardiomyopathy with a significant improvement in left ventricular wall motion following revascularization for a honeycomb-like structure of the left anterior descending artery: a case report

**DOI:** 10.1093/ehjcr/ytag265

**Published:** 2026-04-15

**Authors:** Hiroshi Yoshikawa, Yoshihiro Hanyu, Yuko Onishi, Taishi Yonetsu, Tetsuo Sasano

**Affiliations:** Department of Cardiology, Hiratsuka Kyosai Hospital, 9-11 Oiwake, Hiratsuka, Kanagawa 254-8502, Japan; Department of Cardiology, National Hospital Organization Disaster Medical Center, 3256 Midoricho, Tachikawa, Tokyo 190-0014, Japan; Department of Cardiology, Hiratsuka Kyosai Hospital, 9-11 Oiwake, Hiratsuka, Kanagawa 254-8502, Japan; Department of Cardiovascular Medicine, Institute of Science Tokyo, 1-5-45 Yushima, Bunkyo-ku, Tokyo 113-8510, Japan; Department of Cardiovascular Medicine, Institute of Science Tokyo, 1-5-45 Yushima, Bunkyo-ku, Tokyo 113-8510, Japan

**Keywords:** Case report, Honeycomb-like structure, Ischaemic cardiomyopathy, Myocardial viability

## Abstract

**Background:**

The honeycomb-like structure is an uncommon cause of myocardial ischaemia, detectable through high-resolution intracoronary imaging. Although percutaneous coronary intervention (PCI) for honeycomb-like structure has shown favourable outcomes, the relationship between PCI for honeycomb-like structure and subsequent improvement in cardiac function remains poorly understood.

**Case summary:**

A 58-year-old male was referred to our hospital for exertional dyspnoea. He was diagnosed with heart failure, and echocardiography revealed a reduced left ventricular ejection fraction (LVEF) of 24%, with particularly severe hypokinesis observed in the left anterior descending artery (LAD) territory. Cardiac magnetic resonance imaging showed non-transmural late gadolinium enhancement predominantly in the endocardial layer of the anterior LV wall. Coronary angiography demonstrated diffuse moderate stenosis in the LAD, with fractional flow reserve (FFR) of 0.29, and optical coherence tomography revealed a honeycomb-like structure. Two everolimus-eluting stents were implanted in the LAD. Following the PCI, significant recovery of LV wall motion including the anterior and apical segments was observed, with LVEF increasing from 24% to 46%. The 1-year follow-up showed no worsening of heart failure.

**Discussion:**

This is the first documentation of an ischaemic cardiomyopathy showing significant recovery of cardiac function after PCI targeting honeycomb-like structure. As reported in previous studies focusing on honeycomb-like structure, this case also exhibited angiographic underestimation of stenosis and low FFR value reflecting the true haemodynamic severity. The pathogenesis of honeycomb-like structure—thrombus formation and spontaneous recanalization—may have triggered ischaemic preconditioning, resulting in the development of hibernating myocardium, which could explain the viable myocardium in this case.

Learning points

- When ischaemic cardiomyopathy is suspected but angiographic stenosis is insufficient to explain the cause, a honeycomb-like structure should be considered.
- To accurately diagnose a honeycomb-like structure, coronary physiological assessment and intravascular imaging should be actively used.
- Even in severe left ventricular dysfunction, a honeycomb-like structure may indicate viable myocardium, and revascularization could improve wall motion.

## Introduction

The honeycomb-like structure is a rare intracoronary finding associated with myocardial ischaemia, identifiable using high-resolution intracoronary imaging techniques. This structure likely represents coronary thrombus formation and spontaneous recanalization.^1–4^ Although percutaneous coronary intervention (PCI) for honeycomb-like structure has shown favourable clinical outcomes,^5^ no previous reports have demonstrated that revascularization targeting honeycomb-like structure in patients with impaired cardiac function results in notable recovery of left ventricular ejection fraction (LVEF). Herein, we present a case of ischaemic cardiomyopathy with marked LV function improvement after PCI for honeycomb-like structure in the left anterior descending artery (LAD).

## Summary figure

**Table:** 

Initial presentation	A 58-year-old man was referred to our hospital for exertional dyspnoea. BNP was elevated to 945 pg/mL, and he was diagnosed with heart failure with reduced ejection fraction (HFrEF) (LVEF 24%). Severe hypokinesis of the anterior wall on echocardiography suggested ischaemic cardiomyopathy in the LAD territory.
During hospitalization	He was treated with intravenous dobutamine and diuretics, as well as guideline-directed medical therapy, and was discharged on Day 13.
1 month later	Cardiac magnetic resonance imaging (MRI) showed late gadolinium enhancement (LGE) in the anterior wall with a transmural extent of 25%–50%. Echocardiography demonstrated no significant improvement in LV wall motion.
3 months later	Coronary angiography revealed diffuse 50%–75% LAD stenosis with a fractional flow reserve (FFR) of 0.29 and optical coherence tomography (OCT) identified a honeycomb-like structure. PCI was performed with implantation of two everolimus-eluting stents.
4 months later	LVEF had improved to 46%, with marked recovery of anterior wall motion.
1 year later	BNP decreased to 18 pg/mL. There have been no episodes of worsening heart failure.

## Case presentation

**Figure 1:**
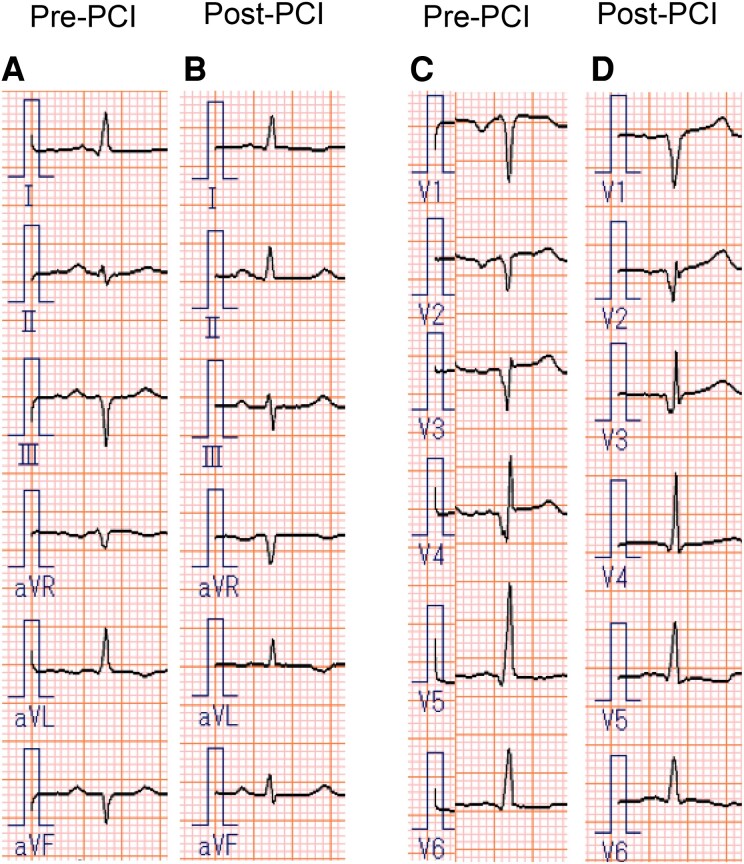
Electrocardiographic changes before and after percutaneous coronary intervention. (*A* and *C*) Pre-intervention electrocardiograms showing abnormal Q waves in leads III, aVF, and V1–V5. (*B* and *D*) Post-intervention electrocardiograms demonstrating resolution of Q waves in leads III, aVF, and V3–V5.

**Figure 2:**
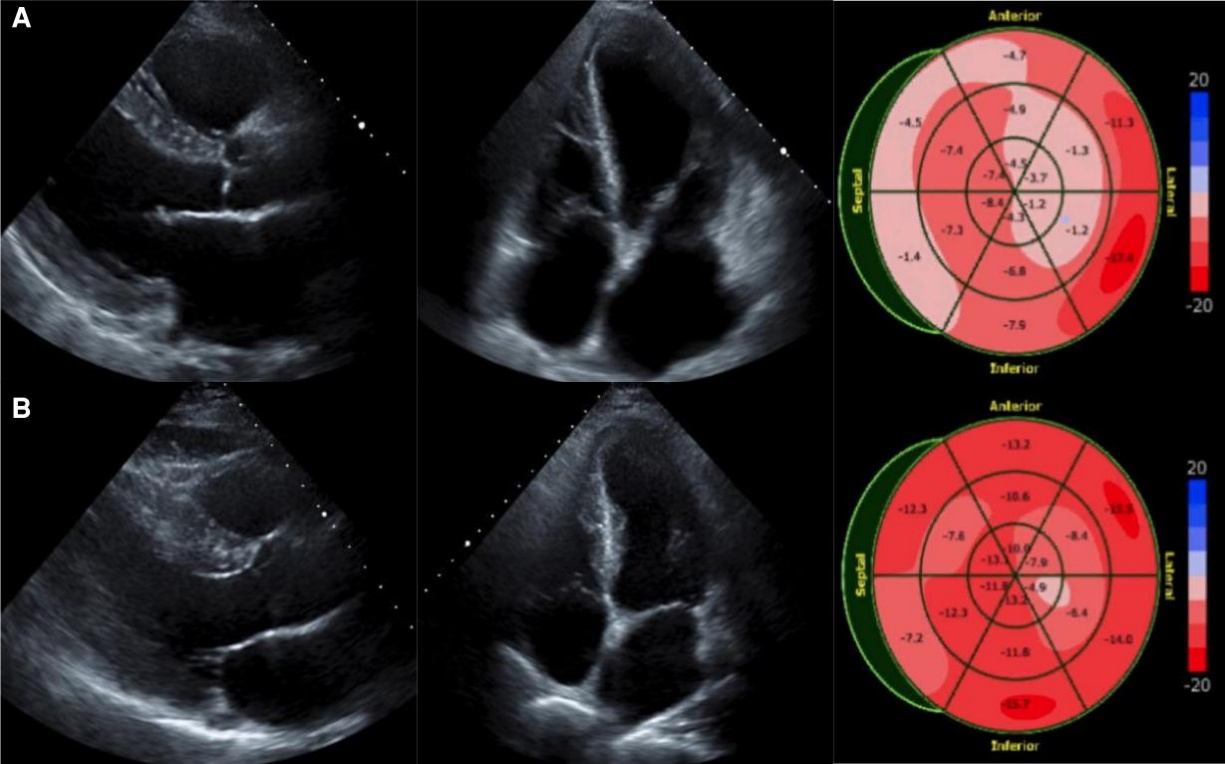
Echocardiographic changes before and after percutaneous coronary intervention. (*A*) Pre-intervention echocardiography. Left ventricular ejection fraction was reduced to 24%, with markedly decreased strain particularly in the left anterior descending artery territory. (*B*) Post-intervention echocardiography. Left ventricular ejection fraction recovered to 46% with wall motion recovery in the left anterior descending artery territory.

**Figure 3:**
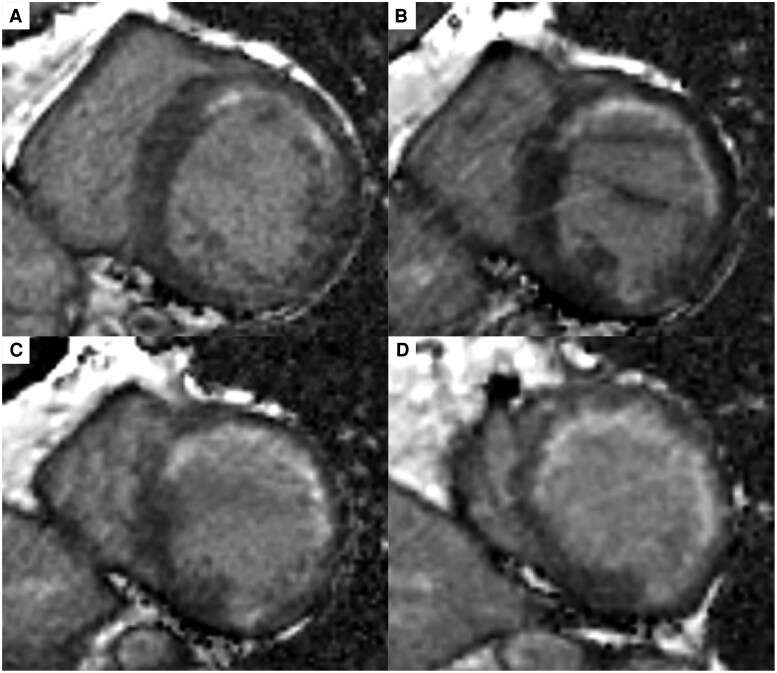
Cardiac magnetic resonance imaging. Subendocardial late gadolinium enhancement was observed in left anterior descending artery territory. Short-axis views of the left ventricle at the basal (*A*), mid-ventricular (*B* and *C*), and apical (*D*) levels.

**Figure 4:**
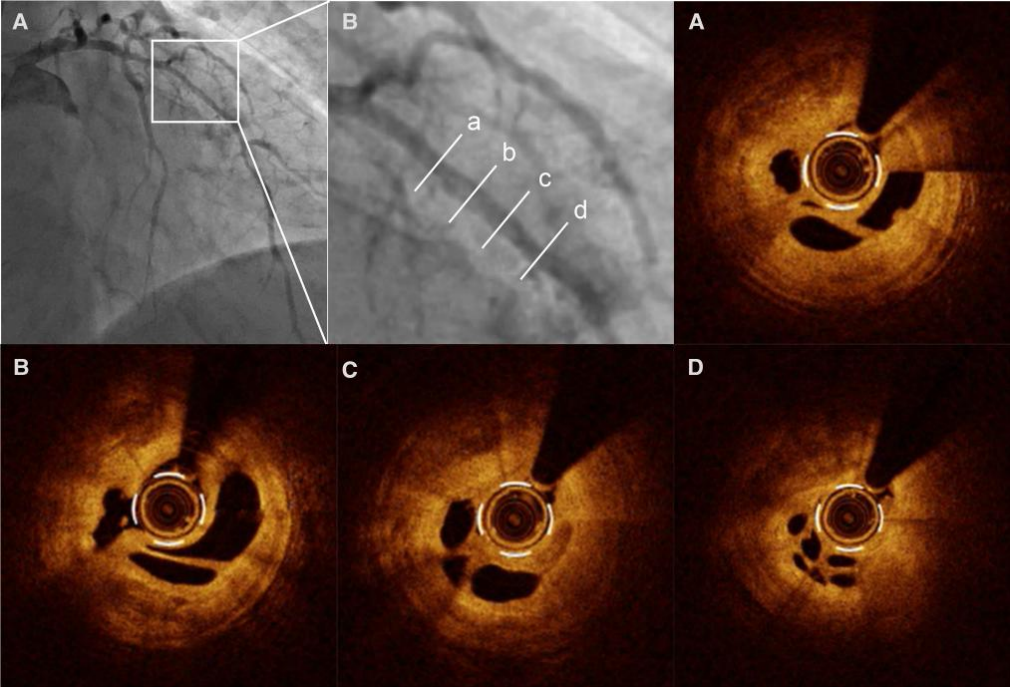
Pre-intervention optical coherence tomography image of the left anterior descending artery. (*A*) Pre-intervention left coronary angiogram. (*B*) A magnified image of the left anterior descending artery. (*A–D*) Optical coherence tomography identified honeycomb-like structure consisting of signal-rich, highly backscattered septa dividing the lumen into multiple small cavities.

**Figure 5:**
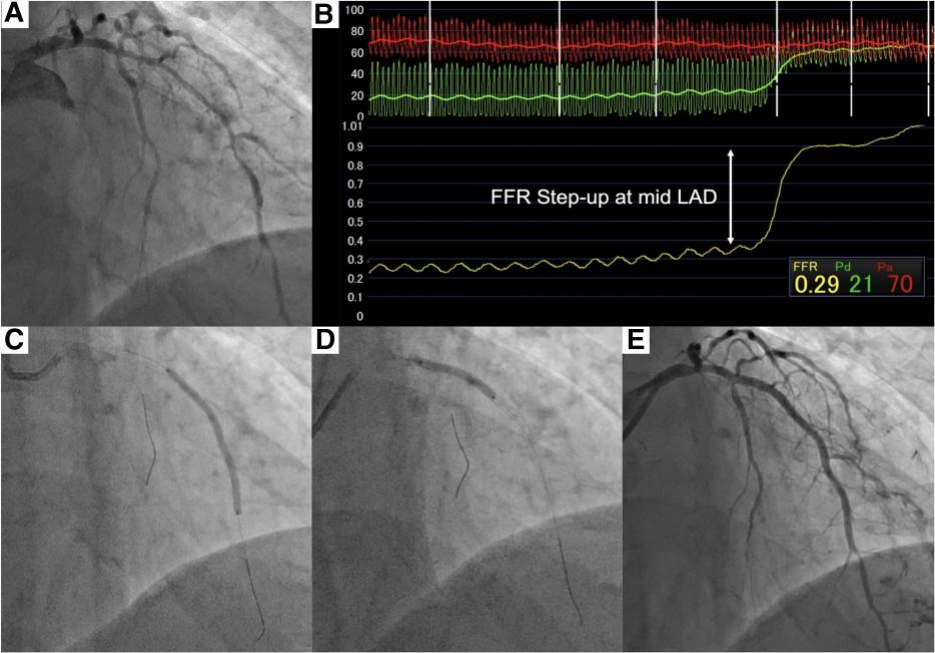
Percutaneous coronary intervention. (*A*) Pre-intervention left coronary angiogram. Diffuse 50%–75% stenosis was observed in left anterior descending artery. (*B*) Fractional flow reserve pullback tracing. The fractional flow reserve was markedly reduced to 0.29, with the largest step-up observed at the mid–left anterior descending artery. (*C* and *D*) Two everolimus-eluting stents were implanted. (*E*) Post-intervention left coronary angiogram.

A 58-year-old male with hypertension was referred to our hospital due to exertional dyspnoea for 2 weeks. His vital signs were as follows: blood pressure, 103/77 mmHg; heart rate, 106 b.p.m.; and oxygen saturation (SpO_2_), 94% on room air. Pulmonary auscultation revealed bilateral wheezing, and cardiac auscultation detected an S3 gallop. Additionally, marked bilateral lower extremity oedema was observed. The electrocardiogram showed sinus tachycardia with abnormal Q waves in leads III, aVF, and V1–V5 (*Figure 1A* and *C*). Laboratory analyses revealed an elevated BNP level of 945 pg/mL, while high-sensitivity cardiac troponin I remained within the normal range. Chest X-ray demonstrated cardiac enlargement and pulmonary oedema, with suspected bilateral pleural effusion. Transthoracic echocardiography revealed LV dilation, reduced LVEF of 24% (*Figure 2A,*  Supplementary material online, *S1*), and severe hypokinesis particularly in the LAD territory, with some areas of myocardial thinning. The patient was diagnosed with HFrEF, with suspected ischaemic cardiomyopathy. Upon hospital admission, continuous intravenous dobutamine infusion and diuretic therapy were initiated. Guideline-directed medical therapy was introduced stepwise, including angiotensin receptor-neprilysin inhibitor, beta-blocker, mineralocorticoid receptor antagonist, and sodium-glucose cotransporter-2 inhibitor. Aspirin was initiated as well, given the strong suspicion of underlying coronary ischaemia. He was discharged on Day 13. Cardiac MRI revealed LGE predominantly in the endocardial layer of the anterior LV wall, with a transmural extent of 25%–50%, suggesting partially preserved myocardial viability (*Figure 3*). Echocardiography demonstrated no significant improvement in LVEF or anterior wall motion at this point. Because the patient developed COVID-19 and had personal scheduling limitations, coronary angiography—although medically warranted at an earlier stage—was deferred for approximately 3 months. The eventual angiography revealed diffuse 50%–75% LAD stenosis, with Thrombolysis In Myocardial Infarction (TIMI) Grade 3 flow maintained (*Figure 5A*). To evaluate the indication of revascularization, we conducted a coronary physiological assessment, revealing a markedly low FFR of 0.29 (*Figure 5B*). On OCT, diffuse fibrous and calcified plaque was observed in the LAD. Additionally, at the site where the largest step-up was observed on FFR pullback, honeycomb-like structure was identified, consisting of signal-rich, highly backscattered septa that divided the lumen into multiple small cavities (*Figure 4*). We suspected that LV dysfunction was attributed to LAD thrombotic occlusion followed by spontaneous recanalization. Given the presence of potentially salvageable myocardium, we decided to perform PCI. Two everolimus-eluting stents (3.0 × 24 mm, 2.5 × 48 mm) were implanted (*Figure 5C* and *D*, Supplementary material online, *S2*), resulting in a post-PCI FFR of 0.83 with no apparent pressure step-up on pullback. Dual antiplatelet therapy with aspirin 100 mg daily and prasugrel 3.75 mg daily was continued for 6 months, and the regimen was then switched to prasugrel monotherapy. Transthoracic echocardiography performed 1 month after PCI demonstrated a significant improvement of LV wall motion including the anterior and apical segments, with the LVEF increasing from 24% to 46% (*Figure 2B,*  Supplementary material online, *S3*). BNP was decreased to 18 pg/mL, patient’s dyspnoea resolved, and no worsening of heart failure occurred during 1-year follow-up.

## Discussion

We observed a marked improvement in LV function following PCI for the LAD lesion involving honeycomb-like structure. Honeycomb-like structure frequently causes haemodynamically significant stenosis with positive FFR results, although angiography often underestimates the degree of stenosis.^6,7^ This discrepancy is likely attributable to multiple contrast-filled channels within honeycomb-like structure visualized on CAG as a single large vessel due to overlapping projections, although the actual main lumen is markedly narrow. In our case as well, although ischaemia in the anterior wall was clinically suspected, CAG revealed only diffuse moderate LAD stenosis. The positive FFR result, followed by the identification of honeycomb-like structure on OCT, reinforced the diagnosis of ischaemic cardiomyopathy.

Importantly, although echocardiography showed severe anterior hypokinesis, LGE on MRI was not transmural, and LV systolic function significantly recovered following revascularization. Electrocardiogram also demonstrated the resolution of Q waves in leads III, aVF, and V3–V5. This clinical course suggests the presence of hibernating myocardium, a state of persistently impaired LV function resulting from chronically reduced coronary blood flow, with the potential for recovery following perfusion improvement.^8,9^ In our case, at the time of the initial CAG, TIMI Grade 3 flow was preserved. This likely allowed a minimal oxygen supply and myocardial viability. Additionally, the pathophysiological mechanisms of honeycomb-like structure may contribute to the development of hibernating myocardium. In the process of honeycomb-like structure development, thrombus formation induces transient yet severe myocardial ischaemia, followed by spontaneous recanalization and long-standing severe stenosis. These brief ischaemia-reperfusion episodes may enhance myocardial resistance to subsequent prolonged ischaemia through ischaemic preconditioning, which is believed to play a crucial role in the formation of hibernating myocardium.^10^ Although the connection between honeycomb-like structure and hibernating myocardium has not been previously reported, their similar pathophysiological characteristics suggest a possible association.

In our case, while LVEF showed little change 1 month after the initiation of optimal medical therapy (OMT), the marked improvement observed after PCI implies that PCI may have offered additional therapeutic value beyond OMT alone. Recently, the REVIVED-BCIS2 trial demonstrated that, among patients with severe left ventricular systolic dysfunction and extensive coronary disease who received OMT, the addition of revascularization by PCI failed to improve prognosis.^11^ There was no significant difference in the improvement of LVEF between the OMT group and the PCI group. However, this trial predominantly enrolled patients in the chronic phase who were largely asymptomatic (New York Heart Association Class I–II). In contrast, the RESTORE EF study enrolled a substantial proportion of acutely ill, severely symptomatic patients and demonstrated improvement in LVEF after PCI.^12^ The clinical profile of the present patient is close to this population, supporting the notion that PCI can be an effective strategy for left ventricular functional recovery in selected patients with ischaemic cardiomyopathy. Clarifying which patient subsets are most likely to benefit from PCI remains a major challenge in future clinical practice and research.

## Lead author biography


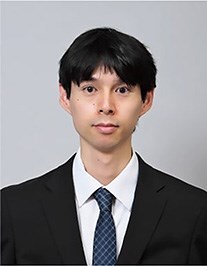


Hiroshi Yoshikawa graduated from Yokohama City University and completed the MD course in 2018. He is currently working as a cardiovascular interventional fellow at Hiratsuka Kyosai Hospital.

## Supplementary Material

ytag265_Supplementary_Data

## Data Availability

The data underlying this article are available in the article and in its online Supplementary material.
