# Supplementary figures and images for: Ischaemic cardiomyopathy with a significant improvement in left ventricular wall motion following revascularization for a honeycomb-like structure of the left anterior descending artery: a case report
Source: Eur Heart J Case Rep. 2026 Apr 15;10(4):ytag265. doi: 10.1093/ehjcr/ytag265 (PMC13128195; doi:10.1093/ehjcr/ytag265)

## Slide 1
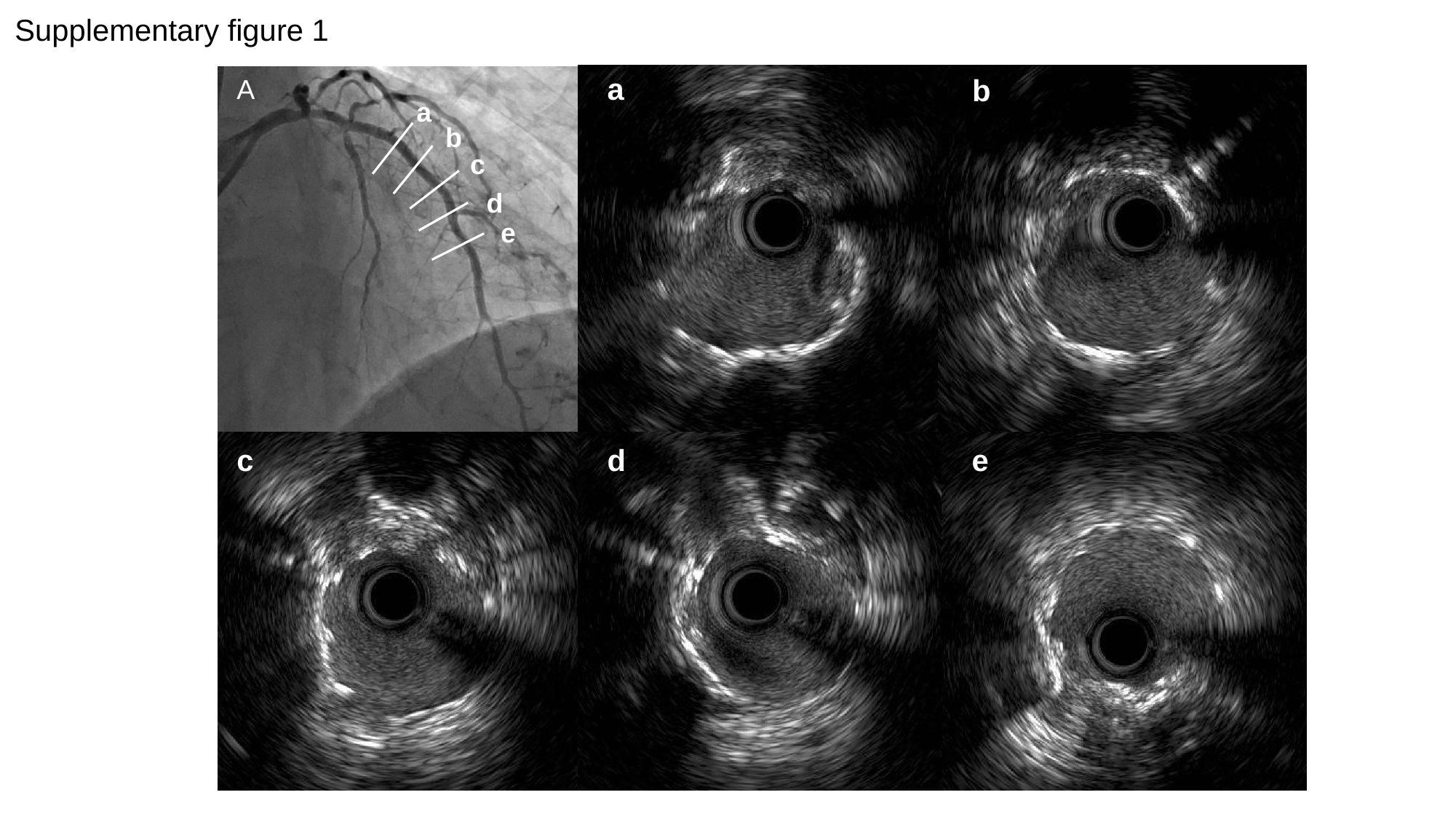

Supplementary figure 1
A
B
a
b
a
b
c
d
e
b
d
e
c

Supplement: ytag265_Supplementary_Data [file ytag265_supplementary_data.zip › Supplementary material S2.pptx]
